# Protective effect of silencing Stat1 on high glucose-induced podocytes injury via Forkhead transcription factor O1-regulated the oxidative stress response

**DOI:** 10.1186/s12860-019-0209-0

**Published:** 2019-07-23

**Authors:** Hongkun Wang, Yanhui Zhang, Fangfang Xia, Wei Zhang, Peng Chen, Guoan Yang

**Affiliations:** 1 Department of Nephrology, The First Affiliated Hospital of Baotou Medical College Inner Mongolia University of Science and Technology, Baotou, China; 2 Department of Nephrology, North Hospital, Baotou, China; 3 Central Laboratory, The First Affiliated Hospital of Baotou Medical College Inner Mongolia University of Science and Technology, No.41 Linyin Road, Kundulun District, Baotou, 014010 Inner Mongolia China; 4 Department of Nutriology, The First Affiliated Hospital of Baotou Medical College Inner Mongolia University of Science and Technology, Baotou, China

**Keywords:** Stat1, FoxO1, Podocyte, HG, Oxidative stress

## Abstract

**Background:**

Podocyte plays an important role in maintaining the integrity and function of the glomerular filtration barrier. Various studies reported that forkhead transcription factor (Fox) O1 played a key role in anti-oxidative signaling. This study aimed to investigate the role of Stat1 in high glucose (HG) -induced podocyte injury.

**Methods:**

Under normal glucose, hypertonic and HG stimulated podocyte conditions, cell counting kit-8 (CCK-8) assay, flow cytometry and western blot and quantitative real-time polymerase chain reaction (qRT-PCR) were respectively carried out to determine cell viability, apoptosis, reactive oxygen species (ROS) production and related genes expressions. We then respectively used silent Stat1, simultaneous silencing Stat1 and FoxO1 and over-expression of FoxO1, to observe whether they/it could reverse the damage of podocytes induced by HG.

**Results:**

High glucose attenuated cell survival and promoted cell apoptosis in MPC-5 cells at the same time, and it was also observed to promote the protein expression of Stat1 and the FoxO1 expression inhibition. Silencing Stat1 could reverse HG-induced podocytes injury. Specifically, siStat1 increased cell viability, inhibited cell apoptosis and attenuated ROS level in a high-glucose environment. Cleaved caspase-3 and pro-apoptosis protein Bax was significantly down-regulated, and anti-apoptosis protein Bcl-2 was up-regulated by siStat1. The antioxidant genes Catalase, MnSOD, NQO1 and HO1 were up-regulated by siStat1. We found that silencing FoxO1 reversed the protective effect of siStat1 on the HG-induced podocytes injury.

**Conclusions:**

Silencing Stat1 could reverse the effects of high glucose-triggered low cell viability, cell apoptosis and ROS release and the functions of Stat1 might be involved in FoxO1 mediated-oxidative stress in nucleus.

## Background

The prevalence of diabetes nephropathy (DN) among diabetic patients is about 20–40% [1]. DN is one of the microvascular complications of diabetes mellitus and is a main cause of end-stage nephropathy [2, 3]. The most common clinical feature of DN is progressive proteinuria [4]. The occurrence of proteinuria is closely related to the integrity of glomerular filtration barrier (GFB), which is composed of capillary endothelial cells, podocytes and a basement membrane between the two [5]. Podocytes are highly differentiated cells that form the outer layer of the filter membrane [6]. Normal morphology and function of podocytes play important roles in proteinuria formation and renal function maintenance [7]. The pathogenesis of DN involved factors such as hemodynamic changes, metabolic disorders, cytokines, inflammatory responses, oxidative stress, autophagy and epithelial-mesenchymal transition (EMT) [8–10]. The occurrence and development of DN is closely related to oxidative stress [11]. Reactive oxygen species (ROS) is mainly synthesized in mitochondria and is the active product of aerobic metabolism. ROS is an important component of oxidative stress reaction. High glucose stimulation can aggravate oxidative stress, leading to a large accumulation of ROS that is beyond the body’s ability to clear the ROS and would therefore exacerbates oxidative stress injury in diabetics [12].

Forkhead transcription factor O1 (FoxO1, FKHR) is a member of the “O” subfamily of the Fox, which also includes three other members, that is, FoxO3a (FKHRL1), FoxO4 (AFX) and FoxO6 [13, 14]. FoxO1 gene is mapped to chromosome 13 and it has 4 functional domains including nuclear localization signal domain (NLS), the transactivation domain (TA), nuclear export signal (NES) and forkhead domain (FKH) [15–17]. FoxO1 is widely distributed in vivo*,* and as a key regulatory factor, it plays an important role in regulating glycolipid metabolism, transcriptional translation, antioxidant stress and cell cycle in the transcriptional process [18–20]. FoxO transcription factors are key mediators of oxidative stress and are activated by various cellular stress stimuli [19]. Recent studies have found that Fox protein family, such as FoxO1, FoxO3, FoxO4, were associated with oxidative stress [21–23].

Stat is a cytoplasmic protein that binds to target gene regulatory region DNA, and it regulates cell transcription. Seven members (Stat1, 2, 3, 4, 5α, 5β and 6) of mammalian Stat protein family are mainly composed of N-terminal oligomerization domain (NT), DNA binding domain (DBD), Src homology 2 domain (SH2) and C-terminal transcriptional activation region (TAD) [24, 25]. Stat1, the first member discovered in the family of Stat proteins, was corroborated to have the function of regulating physiological process, for example, immune response, proliferation, apoptosis and cell survival [26–28]. Recently, Stat1 was also found to act as a tumor inhibitor in liver cancer, esophageal squamous cell carcinoma, breast cancer and colorectal cancer [29–31].

In this study, we aim to investigate the role of Stat1 in high glucose-induced podocytes injury, and whether this effect was related to FoxO1 mediated oxidative stress.

## Methods

### Cell culture

Immortalized mouse podocyte MPC-5 cell was obtained from Research Facilities of PUMCH Cell Bank (Beijing, China). Undifferentiated MPC-5 cells were cultured in DMEM medium (Gibco, Thermo Fisher, USA) with 10 U/ml mice recombinant γ- interferon (Peprotech Inc., USA) in an incubator with 5% CO_2_ at 33 °C. Podocytes were cultured for 2 weeks in the DMEM medium with mouse recombinant γ- interferon at 37 °C to induce cell differentiation and maturation.

### Immunofluorescence

The podocytes were plated in a 24-well plate. When 40% confluent, the cells were fixed, infiltrated and blocked. Each slide was incubated with anti-nephrin (ab216341, 1:500, Abcam, USA) and anti-podocin (ab50339, 1:500, Abcam, USA) at 4 °C overnight. Then the slides were washed three times with PBS and incubated with Alexa Fluor 488 labeled goat anti-rabbit IgG (A0423, 1:100, Beyotime, Shanghai, China) for 45 min at room temperature. Finally, cells were observed under a confocal laser scanning microscope (Olympus, FV 1000, Center Valley, PA, USA).

### Cell counting kit-8 (CCK-8)

In the presence of electron-coupled carrier 1-methoxy PMS, 2-(2-methoxyl-4-nitrobenzene)-3-(4-nitrophenyl)-5-(2, 4-disulfonylbenzene)-2 h-tetrazole monosodium salt can be reduced by dehydrogenase in mitochondria to produce soluble orange-yellow formazan. The color degree is inversely proportional to cytotoxicity and the color of CCK-8 is closed to the medium with phenol red. Cells were seeded in 96-well plate in DMEM medium at a density of 1 × 10^4^ cells/well and disposed with different regents. Next, 10 μl CCK-8 were added into the cells for additional 2 h at 37 °C in humidified 5% CO_2_. The optical density (OD) was measured at 450 nm wave (Thermo Fisher, Massachusetts, USA).

### Carboxyfluorescein diacetate succinimidyl ester (CFSE) assay

The detached cell suspension of 10 × 10^6^ cells/ml was incubated with 5 μM CFSE (C1031, Beyotime, Shanghai, China) in PBS for 15 min at 37 °C. Followed by washing with PBS for two times, the CFSE-labeled target cells were re-suspended and incubated for 48 h at 37 °C and 5% CO_2_. The fluorescence intensity was measured using a FACSCalibur™ cytometer (BD Biosciences, San Jose, CA, USA).

### Cell transfection

Silencing/over-expressing FoxO1 (sc-425179), silencing Stat1 (sc-44124) and empty control plasmids (sc-37007) were purchased from Santa Cruz Biotechnology (Santa Cruz, CA, USA). MPC-5 cells were seeded in 6-well plate (1.0 × 10^5^) for 24 h prior to transfection. Transient transfection was operated using lipofectamine 3000 (Invitrogen, USA) according to standard protocol. A total of 20 μM si−/over-expressing-RNA and empty control plasmids were respectively transfected using 3.75 μl lipofectamine 3000 diluted in Opti-MEM and incubated at room temperature for 15 min. After being cultured for 6 h, the cells was transferred into DMEM medium containing 10% FBS and maintained for another 48 h.

### Flow cytometry

Cells apoptosis was assessed by flow cytometry with Annexin V-FITC/PE staining kit (Meilun Biotechnology Co., LTD, Dalian, China). Cells were washed twice using washing buffer, and the suspension was cultured with Annexin V and propidium iodide (PI, Yeasen Biotechnology Co., Ltd., Shanghai, China) in the dark at 25 °C for 15 min. Binding buffer was added to each well. The samples were analyzed by flow cytometry. Cells were identified by Annexin V-FITC/PI double fluorescent staining: unlabeled viable cells, PI stained cells (necrotic cells), Annexin V-FITC bounded cells (early apoptotic cells) and double-labeled cells (late apoptotic cells).

The Intracellular ROS levels were measured using 2, 7- dichlorodi- hydrofluorescein diacetate (DCFH-DA, Sigma-Aldrich, St Louis, MO, USA) by a flow cytometer. Cells were seeded in a 6-well plate. The cells were exposed to reagents for 24 h when cell confluence reached 70–80%. 10 μM DCFDA containing 1 ml phosphate buffer saline (PBS) were added to the cells at room temperature for 20 min, and the resulting DCF fluorescence was measured by flow cytometry [27].

### Quantitative real-time polymerase chain reaction (qRT-PCR)

Stat1, FoxO1, Catalase, MnSOD, NQO1 and HO1 were detected by performing qRT-PCR in different groups. Total RNA was isolated from cultured MPC-5 cells by Trizol (Invitrogen, USA) according to the manufacturer’s protocol. Reverse transcription was performed with OrimeScript™ RT reagent kit (TaKaRa, Otsu, Shiga, Japan) at 37 °C for 15 min and at 98 °C for 5 min. cDNA was amplified using SYBR Fast qPCR Mix (Invitrogen, USA) and cycle was set as follows: a pre-denaturation at 95 °C for 10 min, followed by 40 cycles of denaturation at 95 °C for 15 s, annealing/elongation at 60 °C for 1 min. All primers were assisted in the synthesis (Sangon Biotech, Shanghai, China). The primer sequences were listed in Table 1. Amplified products were electrophoresed through 2% agarose gels. The amount of RNA was calculated using the 2^-ΔΔCT^ method, and GAPDH served as an internal control [32].

**Table 1:** Primers used in qRT-PCR

Gene	Primer	Sequence
Stat1	Forward Reverse	5′-GAACTTACCCAGAATGCC −3′ 5′-CTTTCCACCACAAACGAG − 3’
FoxO1	Forward Reverse	5’-CAGCAAATCAAGTTATGGAGGA − 3′ 5′-CTGAGAGGAGGGGTGTTACTAT − 3’
Catalase	Forward Reverse	5’-GAATTCGTTAATAAAGAT − 3′ 5′-GTCGACTTACTTTTTCTTTTTT − 3’
MnSOD	Forward Reverse	5’-GGCCAAGGGAGATGTTACAA − 3′ 5′-GCTTGATAGCCTCCAGCAAC-3’
NQO1	Forward Reverse	5’-CAGCCAATCAGCGTTCGGTA-3′ 5′-CTTCATGGCGTAGTTGAATGATGTC-3’
HO1	Forward	5’-TGCAGGTGATGCTGACAGAGG-3’
	Reverse	5’-GGGATGAGCTAGTGCTGATCTGG-3’
GAPDH	Forward Reverse	5’-TGTGTCCGTCGTGGATCTGA-3′ 5′-TTGCTGTTGAAGTCGCAGGAG-3’

### Western blotting analysis

Cellular proteins were extracted from cultured MPC-5 cells by RIPA lysis buffer (Thermo Scientific, Pierce, Rockford, IL, USA). The concentration of proteins was determined using Bradford method (Amresco, USA). Aliquots of supernatant containing proteins were mixed with loading buffer, and the samples were subjected to 12% SDS-PAGE gel. Next, the resolved proteins were transferred onto a polyvinylidene difluoride membrane, and blots were blocked in 1% milk, TBS, 0.1% Tween-20. Proteins were incubated with following primary antibodies: rabbit anti-Stat1 antibody (phospho S727, ab109461, 1:1000, Abcam, USA), anti-FoxO1 antibody (Ab-256, SAB4300410, 1:1000, Merck, Germany); anti-cleaved caspase-3 antibody (ab2302, 1:1000, Abcam, USA), anti-Bcl-2 (ab32124, 1:1000, Abcam, USA), anti-Bax (ab32503, 1:1000, Abcam, USA); anti-Catalase (ab16731, 1:2000, Abcam, USA), anti-MnSOD (orb94946, 1:1000, Biorbyt, Britain), anti-NQO1 antibody (ab34173, 1:1000, Abcam, USA), anti-HO1 antibody (SMC-131D-HRP, 1:1000, StressMarq, Canada); anti-Lamin B1 antibody (ab16048, 1:1000, Abcam, USA), anti-Tubulin antibody (ab7291, 1:1000, Abcam, USA) and anti-GAPDH antibody (ab9485, 1:2500, Abcam, USA). The proteins were then washed in TBST for 10 min and incubated with the HRP-conjugated goat anti-Rabbit IgG (Protein tech, USA) as secondary antibodies. The blots were developed with enhanced chemiluminescent (ECL; Thermo Fisher Scientific, Inc.). The protein signal of immunoblot analysis was developed using NBT/BCIP system (Roche, Switzerland). The quantification of proteins expressions of were performed by using Quantity one (Bio-Rad, USA).

### Statistical analysis

Statistical analysis was detected by Prism Graphpad version 6.0 software. All data were presented as mean ± standard deviation (SD). Differences were performed using one-way analysis of variance (ANOVA) following Turkey’s multiple comparison. A *p* < 0.05 was considered as statistically significant.

## Results

### High glucose affects cell viability and induces cell apoptosis

**Fig. 1:**
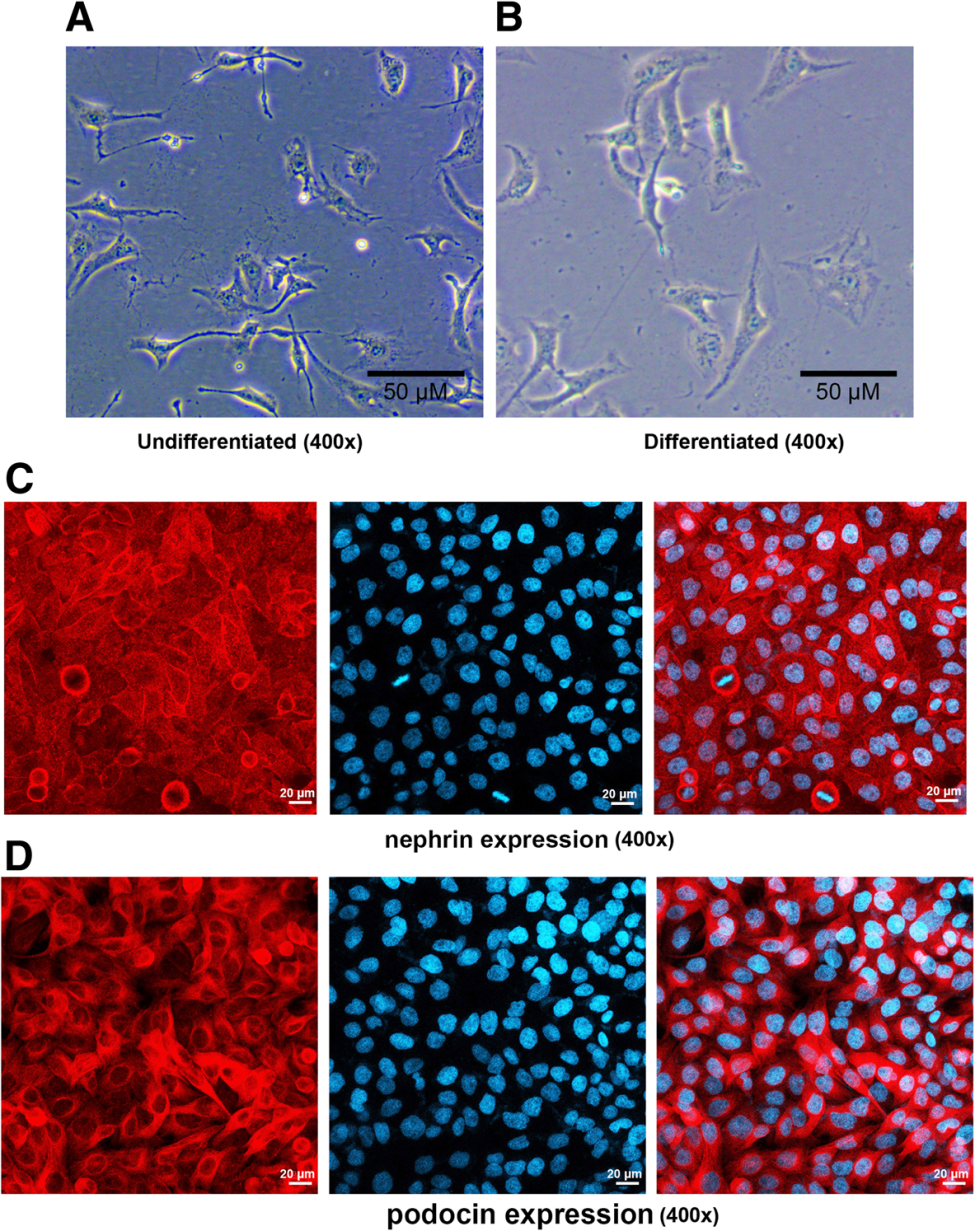
Morphological observation of MPC-5 cells (400×). **a** Under the condition of undifferentiated. **b** Under the condition of differentiated. **c** Immunofluorescence was used to detect the nephrin expression in the cytoplasm of podocytes. **d** Immunofluorescence was used to detect the podocin expression in the cytoplasm of podocytes

The MPC-5 cells were observed by an inverted microscope. Cells show signs of fusiform or triangular under the condition of proliferation with γ-interferon at 33°C (Fig. 1a). After being passaged, the cells ceased to proliferate, and they grew larger, gradually differentiated and matured. The podocytes began to protrude from cytoplasm, forming of the coarse main protrusions and finger-like secondary foot processes (Fig. 1b). Moreover, nephrin and podocin were expressed in the cytoplasm of podocytes (Fig. 1c and d).

**Fig. 2:**
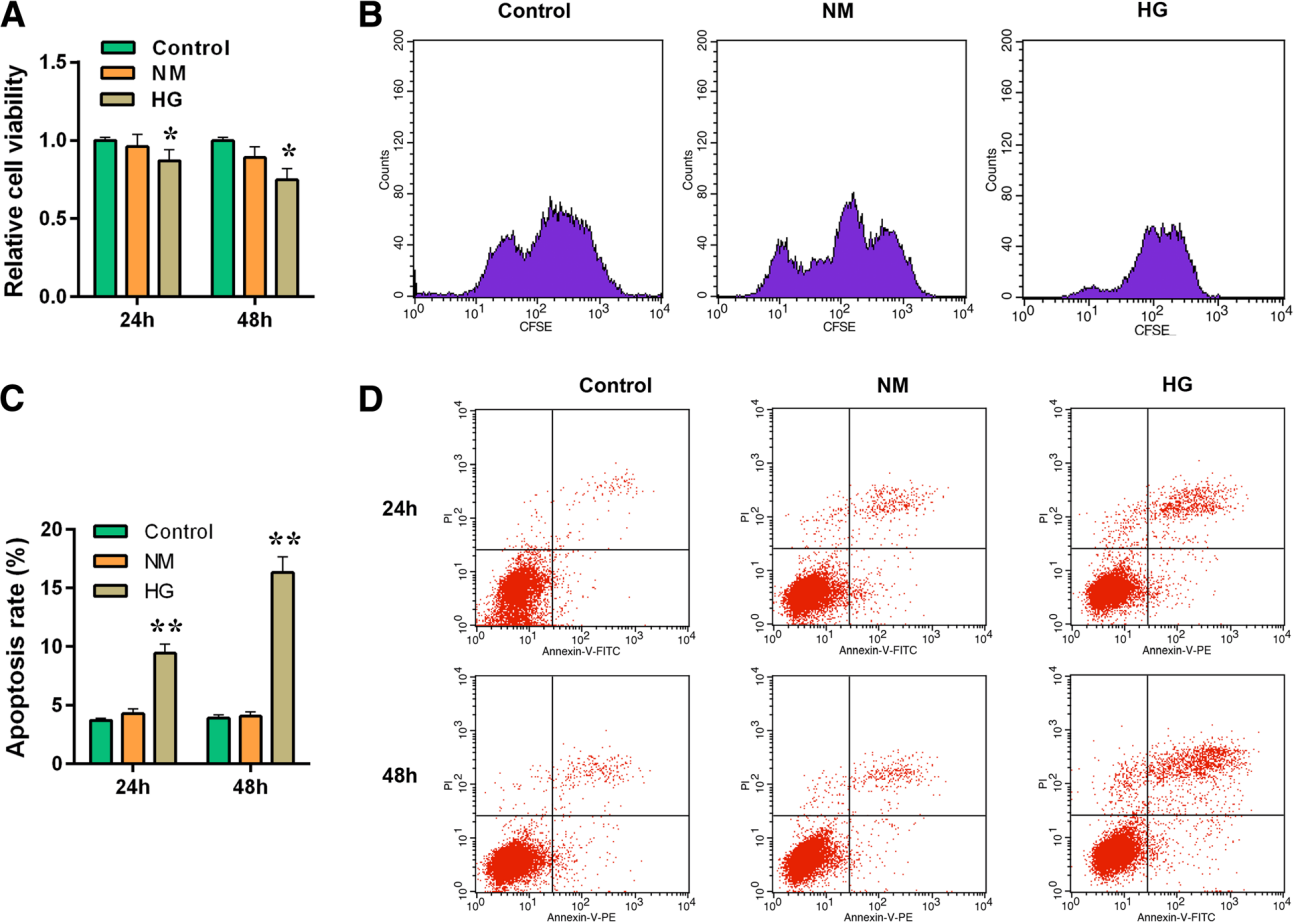
The effect of high glucose stimulation on cell survival and apoptosis in MPC-5 cells. The experiments were divided into three groups, which were control (5.5 mmol/L normal glucose), NM (5.5 mmol/L glucose+ 24.5 mmol/L mannitol) and HG (30 mmol/L glucose). **a** Cell viability was detected for 24 h and 48 h by CCK-8 assay. **b** CFSE assay was used to detect cell survival. **c** Relative apoptosis rate showed as bar diagrams. **d** Apoptosis levels were detected for 24 h and 48 h by flow cytometry. Data were expressed as mean ± SD from three independent experiments. (*Compared with control, *P < 0.05, **P < 0.01)

**Fig. 3:**
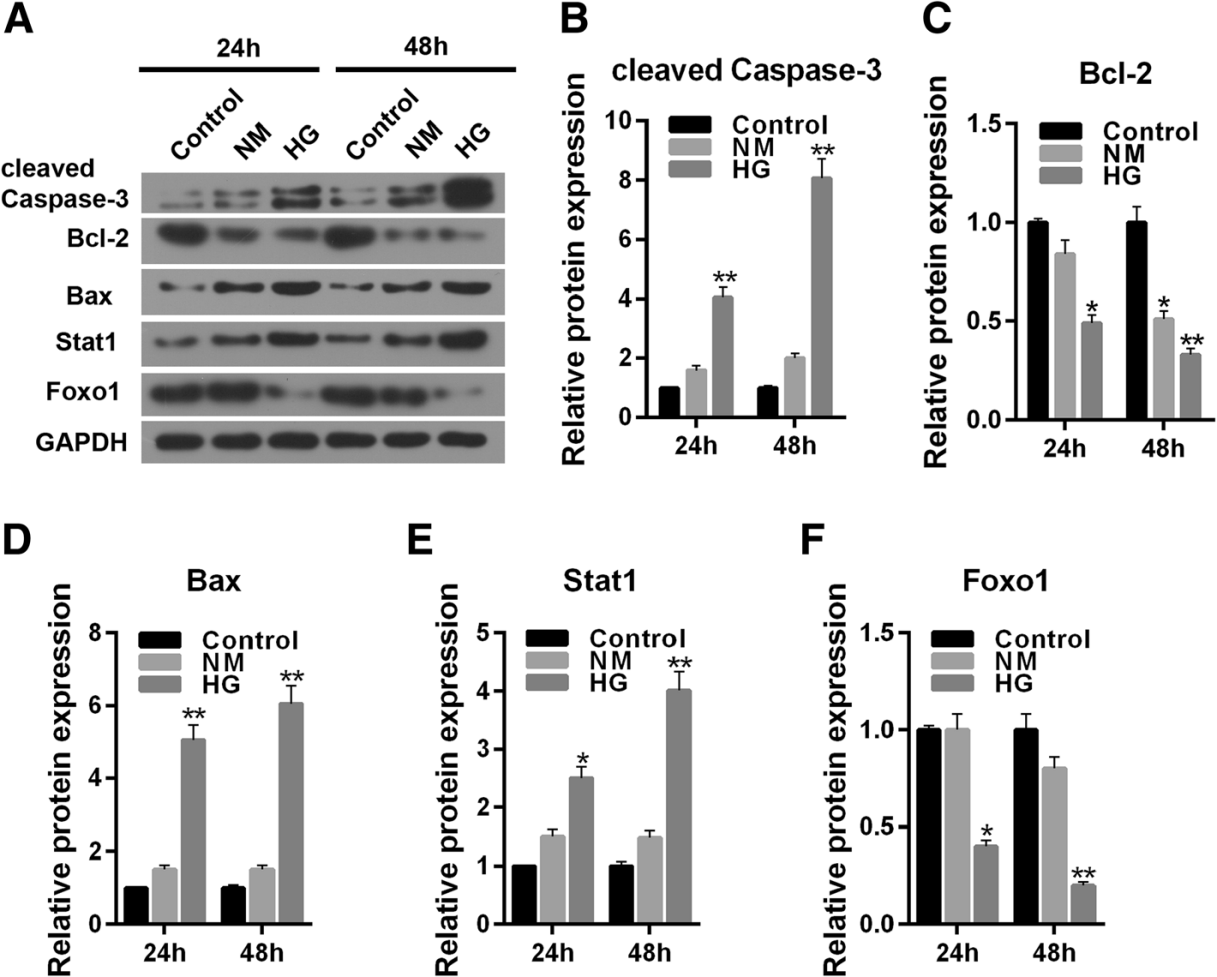
High glucose affects the protein expression of apoptosis related, Stat1 and FoxO1 in MPC-5 cells. The experiments were divided into three groups, which were control (5.5 mmol/L normal glucose), NM (5.5 mmol/L glucose+ 24.5 mmol/L mannitol) and HG (30 mmol/L glucose). **a** The cleaved caspased-3, Bcl-2, Bax, Stat1 and FoxO1 protein levels were detected by western blot in MPC-5 cells. **b** Relative protein expression of cleaved caspased-3 showed as bar diagrams. **c** Relative protein expression of Bcl-2 showed as bar diagrams. **d** Relative protein expression of Bax showed as bar diagrams. **e** Relative protein expression of Stat1 showed as bar diagrams. **f** Relative protein expression of FoxO1 showed as bar diagrams. GAPDH served as an internal control. Data were expressed as mean ± SD from three independent experiments. (*Compared with control, *P < 0.05, **P < 0.01)

To understand the effect of high glucose (30 mmol/L) stimulation in MPC-5 cells, the cell viability and apoptosis were detected by respectively performing CCK-8 and flow cytometry for 24 and 48 h. The result showed that mannitol (24.5 mmol/L) produced limited effect on cell viability (*P* > 0.05) while high glucose exerted an inhibitory effect on cell viability (Fig. 2a, *P* < 0.05), in concert with the results of CFSE assay (Fig. 2b, *P* < 0.05). Moreover, high glucose (30 mmol/L) stimulation aggravated cell apoptosis level both at 24 h and 48 h compared to control (5.5 mmol/L glucose treatment) or NM group (5.5 mmol/L glucose + 24.5 mmol/L mannitol treatement) (Fig. 2c and d, *P* < 0.01). The apoptosis related-genes were also assessed by carrying out western blot. The normal glucose and hypertonic group had no significant differences in cleaved caspase-3, Bcl-2 and Bax proteins level. Compared with control, high glucose could not only clearly up-regulate the protein expressions of cleaved caspase-3 and Bax (Fig. 3a, b and d, *P* < 0.01), but also significantly down-regulated the expression of Bcl-2 protein (Fig. 3a and c, 24 h, *P* < 0.05; 48 h, *P* < 0.01).

### High glucose affects the protein expression of Stat1 and FoxO1

To determine the effect of high glucose on the expressions of Stat1 and FoxO1, the proteins levels were detected by performing western blot. The effects of high glucose on Stat1 and FoxO1 were realized in a time- dependent manner. High glucose increased the protein level of Stat1 significantly (Fig. 3a and e, 48 h, *P* < 0.01) and sharply decreased the expression of FoxO1 protein in 48 h (Fig. 3a and f, *P* < 0.01).

### Silencing Stat1 reverses the effect of reduced cell viability and pro-apoptosis by high glucose

**Fig. 4:**
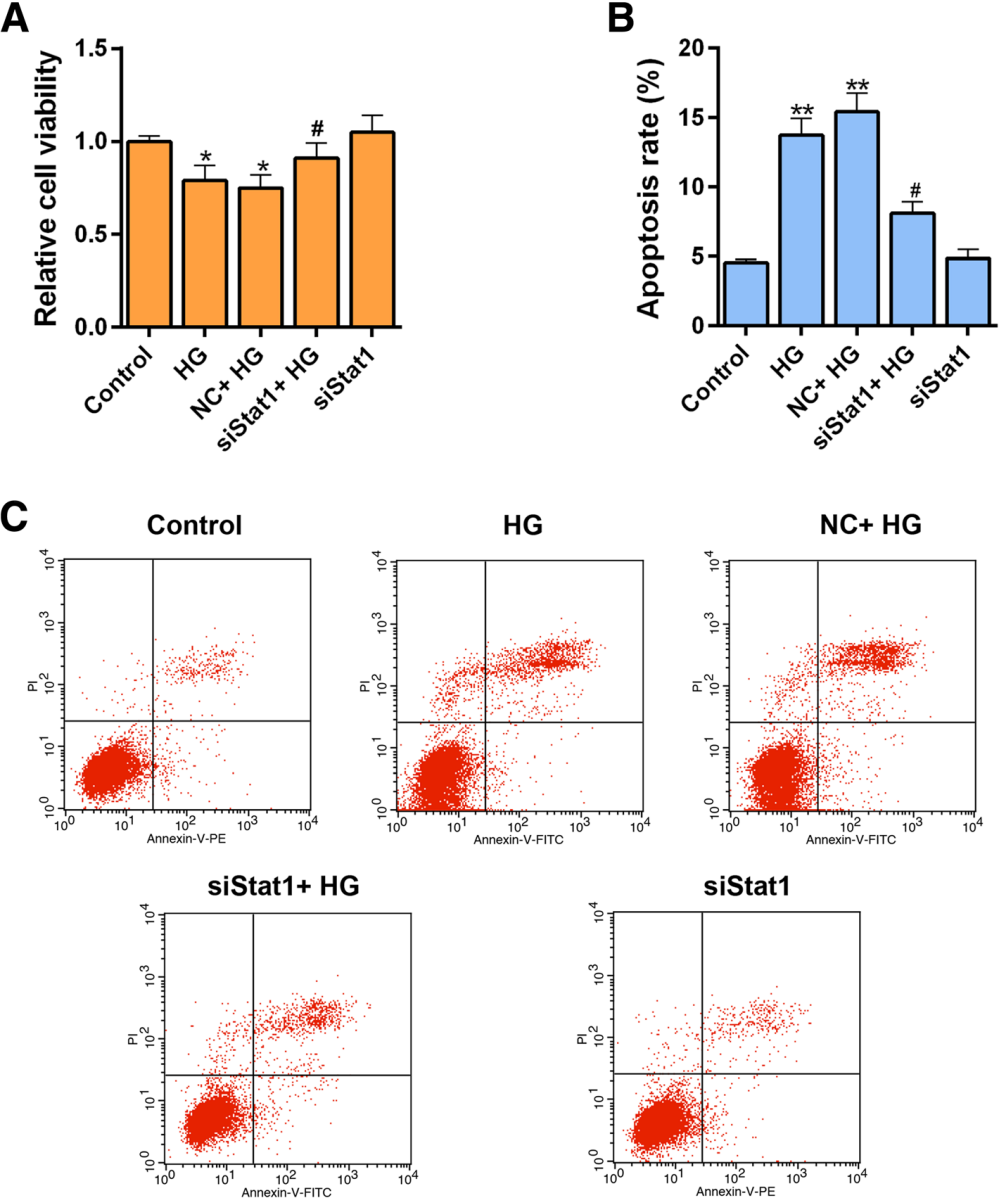
Silencing Stat1 reverses the effect of cell viability and apoptosis by high glucose. The experiments were divided into five groups, which were control, HG (30 μM), NC + HG (Negative siRNA + 30 μM HG), siStat1 + HG (Stat1 siRNA + 30 μM HG) and SiStat1 (Stat1 siRNA). **a** Cell viability was detected by CCK-8 assay. **b** Relative apoptosis rate showed as bar diagrams. **c** Apoptosis levels were detected by flow cytometry. Data were expressed as mean ± SD from three independent experiments. (*Compared with control, ^#^ Compared with HG, */^#^*P* < 0.05, **P < 0.01)

**Fig. 5:**
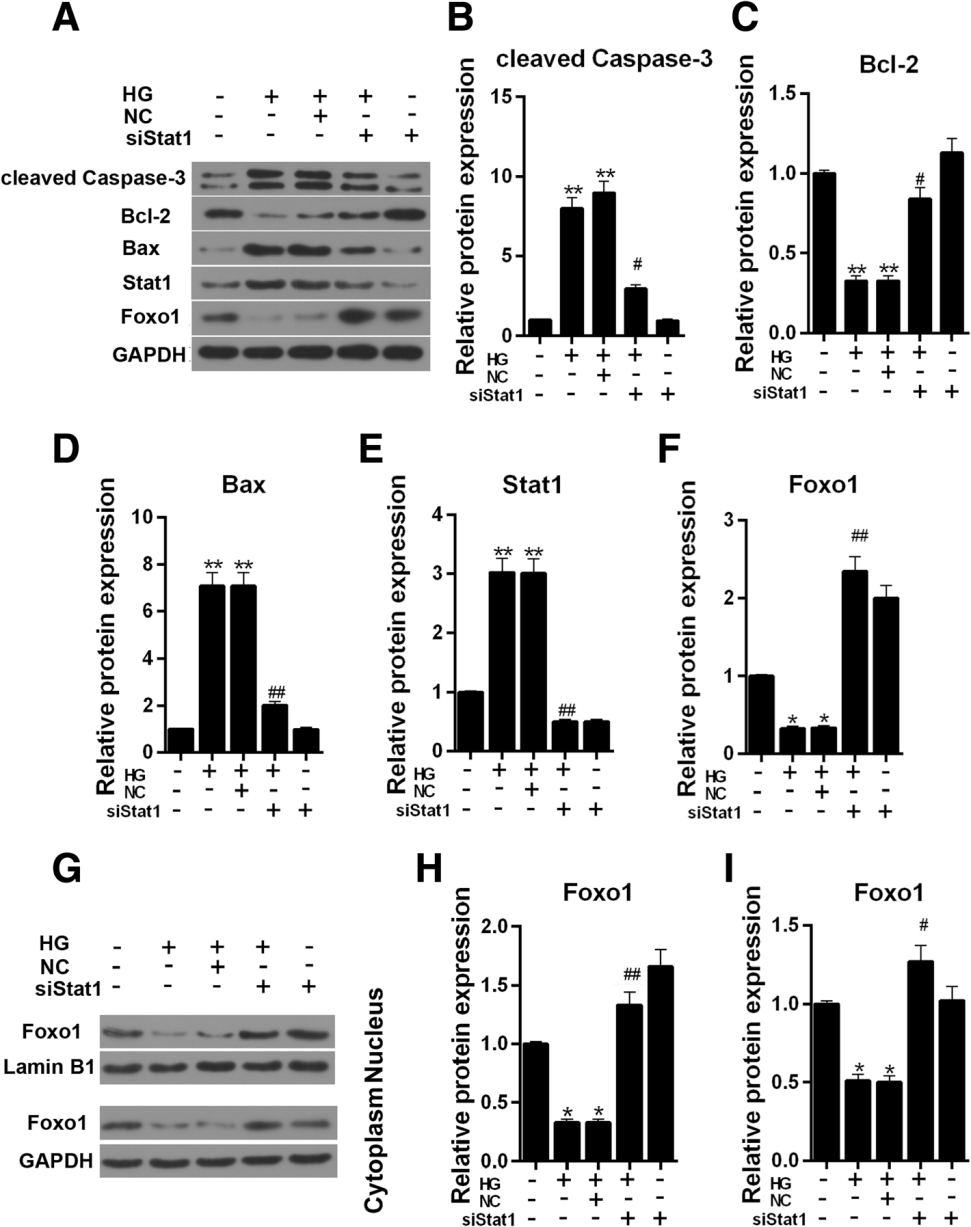
Silencing Stat1affects the protein expression of apoptosis related, Stat1 and FoxO1 in MPC-5 cells. The experiments were divided into five groups, which were control, HG (30 μM), NC + HG (Negative siRNA + 30 μM HG), siStat1 + HG (Stat1 siRNA + 30 μM HG) and SiStat1 (Stat1 siRNA). **a** The cleaved caspased-3, Bcl-2, Bax, Stat1 and FoxO1 protein levels were detected by western blot in MPC-5 cells. **b** Relative protein expression of cleaved caspased-3 showed as bar diagrams. **c** Relative protein expression of Bcl-2 showed as bar diagrams. **d** Relative protein expression of Bax showed as bar diagrams. **e** Relative protein expression of Stat1 showed as bar diagrams. F Relative protein expression of FoxO1 showed as bar diagrams. GAPDH served as an internal control. **g** The protein expressions of FoxO1 in nucleus and cytoplasm, respectively, Lamin B1 served as a nucleus loading control, GAPDH served as a cytoplasm internal control. **h** Relative protein expression of FoxO1 in nucleus showed as bar diagrams. **i** Relative protein expression of FoxO1 in cytoplasm showed as bar diagrams. Data were expressed as mean ± SD from three independent experiments. (*Compared with control, ^#^Compared with HG, */^#^*P* < 0.05, **/^##^*P* < 0.01)

Our study has shown that high glucose increased the expression of Stat1. Silent Stat1 plasmid was transfected into MPC-5 cells to help us explore the role of silencing Stat1. We found that siStat1 could recover the cell viability resulted from high glucose injury (Fig. 4a). The apoptosis assay showed that siStat1 significantly attenuated high glucose- induced apoptosis (Fig. 4b and c, *P* < 0.05). Single siStat1 was found to clearly increase the cell viability and down-regulated the apoptosis compared to group high glucose (Fig. 4a, b and c, Cell viability, *P* < 0.05; Apoptosis, *P* < 0.01). The apoptosis related-genes were also detected using western blot, and we found that pro-apoptosis proteins cleaved caspase-3, and that Bax sharply revealed down-regulation expression in the presence of both siStat1 and high glucose simulation (Fig. 5a, b and d, *P* < 0.05, Bax, *P* < 0.01). However, the Bcl-2 showed the opposite result that siStat1 could significantly increase expression by reversing a high glucose effect (Fig. 5a and c, *P* < 0.05).

### Silencing Stat1 promotes the protein expression of FoxO1 in nucleus

As Fig. 5e shown, in an environment containing high glucose, the protein level of Stat1 was up-regulated (Fig. 5e, *P* < 0.01). The protein expression of Stat1 decreased once siStat1 was added, on the contrary, the protein level of FoxO1 raised (Fig. 5f, *P* < 0.01).

In order to further explore the expression of FoxO1, the protein level of FoxO1 both in cytoplasm and nucleus was tested. Compared to control, the protein level of FoxO1 significantly decreased after HG treatment in cytoplasm and nucleus environment (Fig. 5g, h and i, *P* < 0.05). The expression of FoxO1 was increased in HG + siStat1 in nucleus in compared to HG group (Fig. 5g and h, Control, *P* < 0.05, HG, *P* < 0.01), however, the cytoplasm FoxO1 showed no clear difference to control (Fig. 5g and i, *P* > 0.05).

### Silencing Stat1 reduces ROS level and promotes the expression of antioxidant genes in high glucose injured MPC-5 cells

**Fig. 6:**
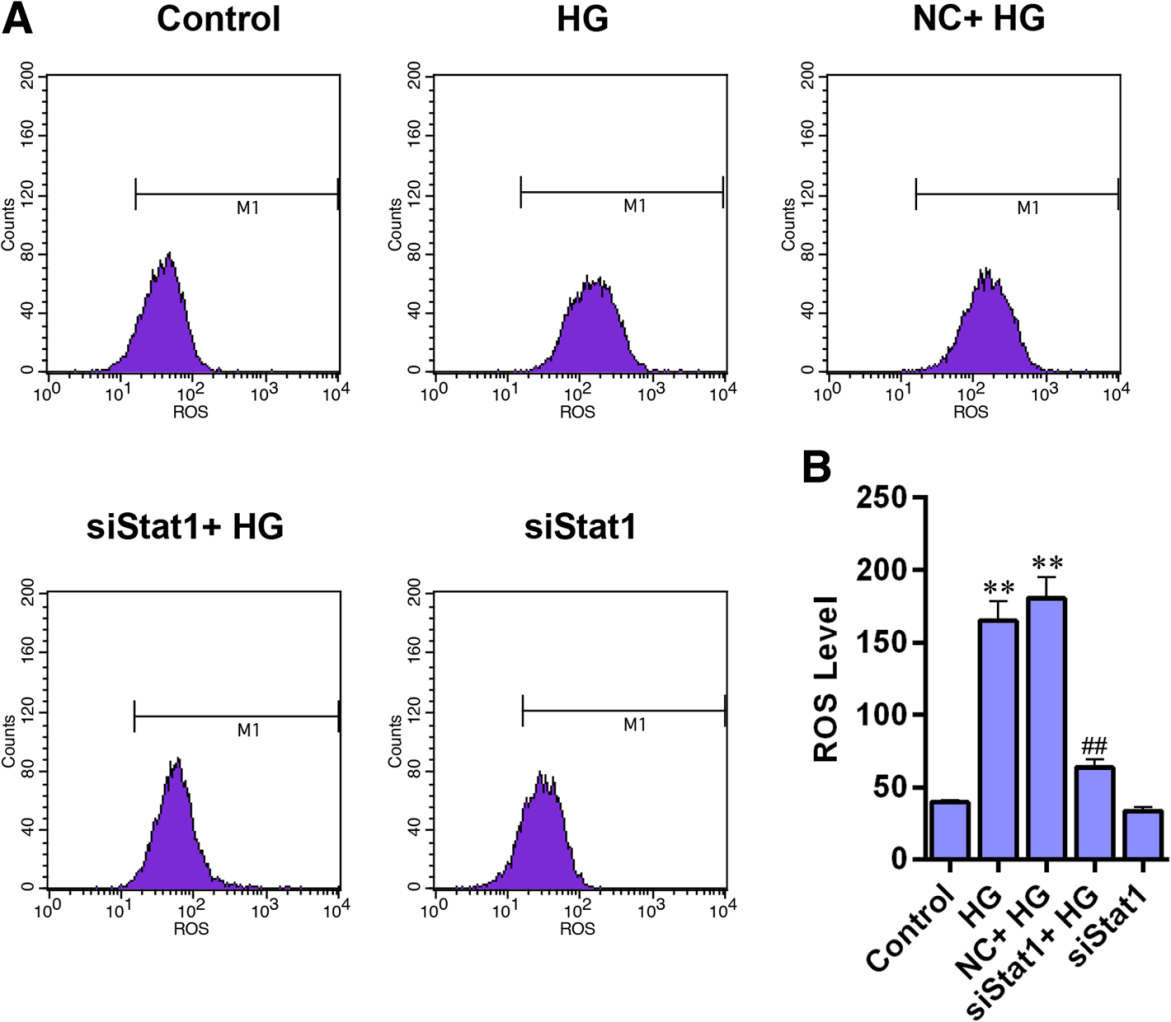
Silencing Stat1 reduces ROS level in high glucose injured MPC-5 cells. **a** ROS levels were detected by flow cytometry. **b** ROS level showed as bar diagrams. Data were expressed as mean ± SD from three independent experiments. (*Compared with control, ^#^Compared with HG, **/^##^*P* < 0.01)

**Fig. 7:**
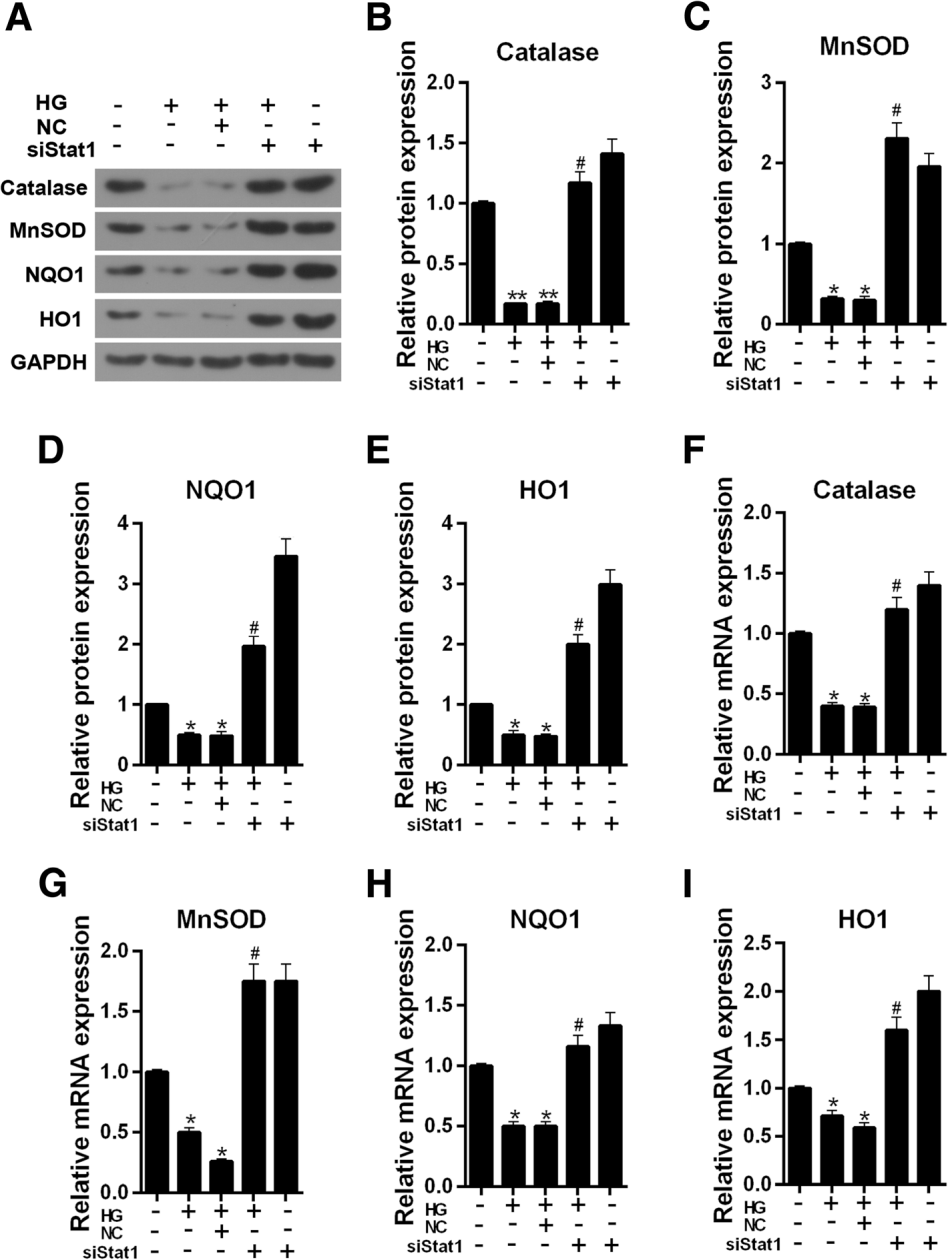
Silencing Stat1 promotes the expression of antioxidant genes in high glucose injured MPC-5 cells. **a** The Catalase, MnSOD, NQO1 and HO1 protein levels were detected by western blot in MPC-5 cells. **b** Relative protein expression of Catalase showed as bar diagrams. **c** Relative protein expression of MnSOD showed as bar diagrams. **d** Relative protein expression of NQO1 showed as bar diagrams. **e** Relative protein expression of HO1 showed as bar diagrams. **f** mRNA expression of Catalase was assessed using qRT-PCR in MPC-5 cells. **g** mRNA expression of MnSOD was assessed using qRT-PCR in MPC-5 cells. **h** mRNA expression of NQO1 was assessed using qRT-PCR in MPC-5 cells. **i** mRNA expression of HO1 was assessed using qRT-PCR in MPC-5 cells. GAPDH served as an internal control. Data were expressed as mean ± SD from three independent experiments. (*Compared with control, ^#^Compared with HG, */^#^*P* < 0.05, **P < 0.01)

The level of ROS was increased noticeably in HG-treated cells, however, the ROS level was alleviated by siStat1 (Fig. 6a and b, *P* < 0.01). We then determined the expressions of antioxidant genes (Catalase, MnSOD, NQO1, HO1) using western blot and qRT-PCR. The western blot analysis showed that these four antioxidant proteins had similar results, which, under a high glucose stimulation, their protein levels was sharply decreased (Fig. 7a-e, Catalase, *P* < 0.01, Others, *P* < 0.05). Single siStat1 and siStat1 with HG significantly enhanced the proteins expression of Catalase, MnSOD, NQO1, HO1 (*P* < 0.01). The mRNA levels of Catalase, MnSOD, NQO1, HO1 had basic consistent results (Fig. 7f-i) with those of proteins.

### Over-expressing FoxO1 decreases high glucose induced apoptosis

**Fig. 8:**
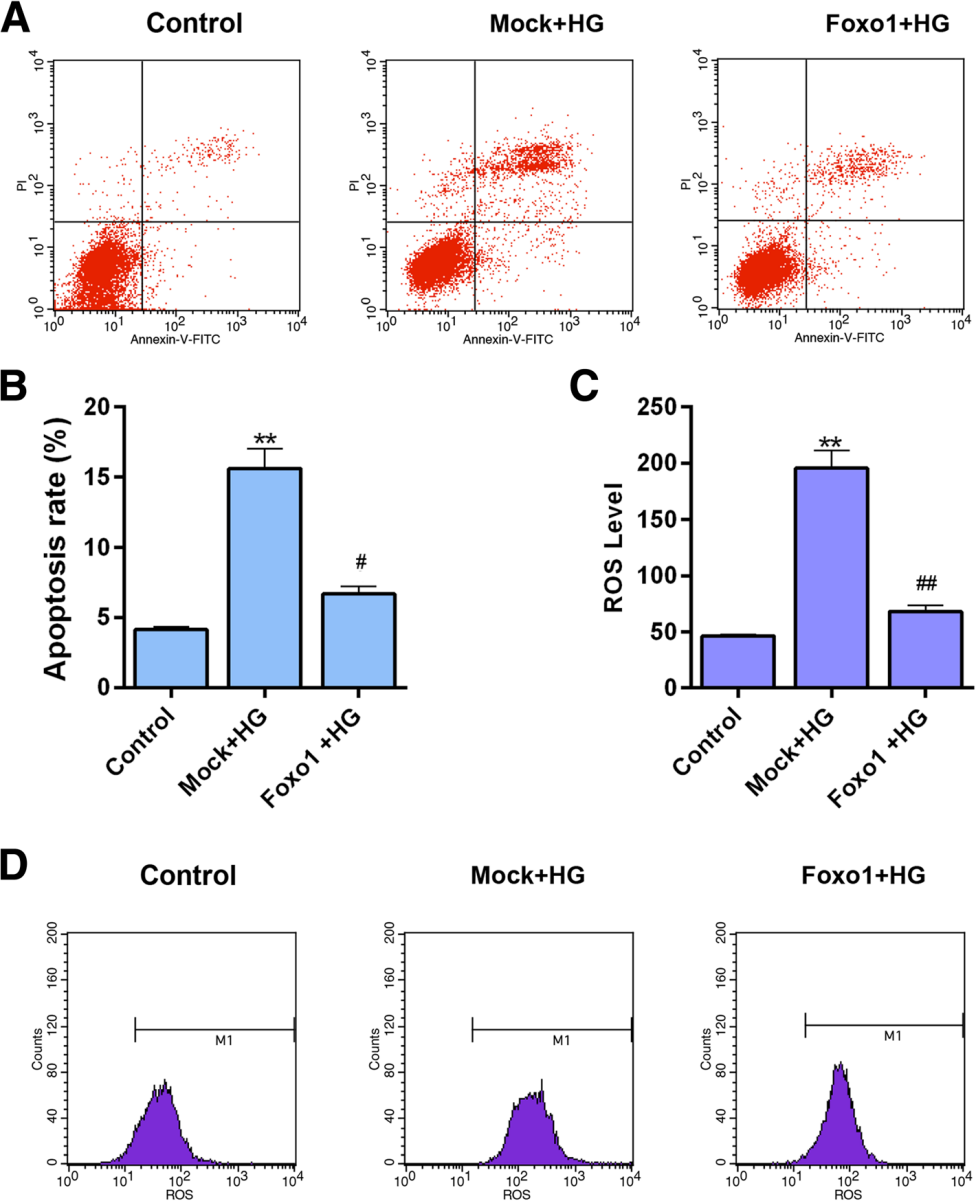
The effect of over-expressing FoxO1 on apoptosis and ROS level. The experiments were divided into three groups, which were control, Mock + HG and over-expressing FoxO1 + HG. **a** Apoptosis levels were detected by flow cytometry. **b** Relative apoptosis rate showed as bar diagrams. **c** ROS level showed as bar diagrams. **d** ROS levels were detected by flow cytometry. Data were expressed as mean ± SD from three independent experiments. (*Compared with control, ^#^ Compared with Mock + HG, ^#^*P* < 0.05, **/^##^*P* < 0.01)

The over-expression of FoxO1 was rendered to help understand the role of FoxO1 in HG injured MPC-5 cells. The apoptosis assay showed that over-expressing FoxO1 could significantly inhibit the HG-caused apoptosis (Fig. 8a and b, *P* < 0.05).

### Over-expressing FoxO1 inhibits ROS production and promotes the expression of antioxidant genes

**Fig. 9:**
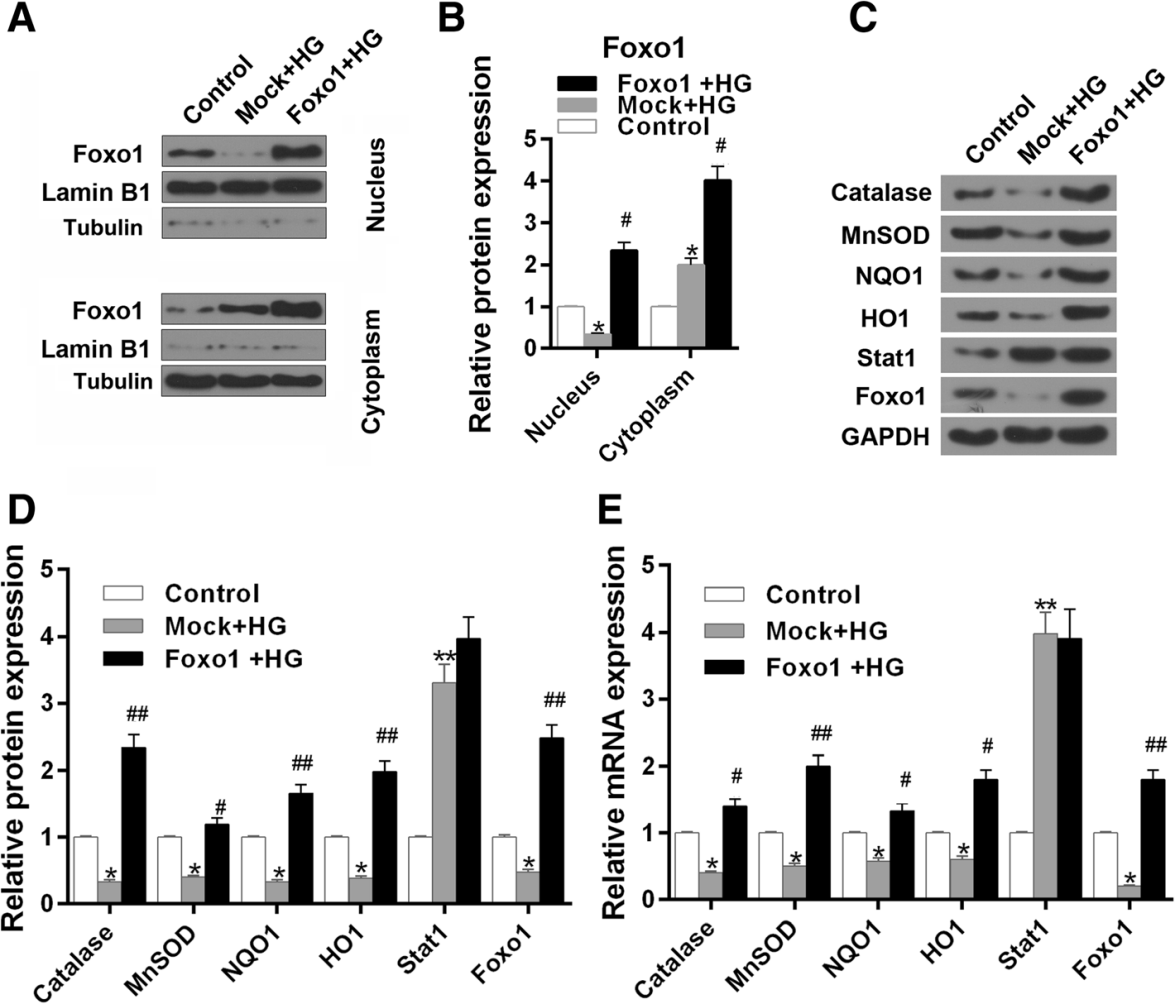
The effect of over-expressing FoxO1 on the expression of antioxidant genes, Stat1 and FoxO1. The experiments were divided into three groups, which were control, Mock + HG and over-expressing FoxO1 + HG. **a** The protein expression of FoxO1 in nucleus and cytoplasm, respectively, Lamin B1 served as a nucleus loading control, Tubulin served as a cytoplasm internal control. **b** Relative protein expressions of FoxO1 in nucleus and cytoplasm showed as bar diagrams. **c** The Catalase, MnSOD, NQO1, HO1, Stat1 and FoxO1 protein levels were detected by western blot in MPC-5 cells. **d** Relative protein expression of Catalase, MnSOD, NQO1, HO1, Stat1 and FoxO1 showed as bar diagrams. **e** mRNA expression of Catalase, MnSOD, NQO1, HO1, Stat1 and FoxO1 were assessed using qRT-PCR in MPC-5 cells. GAPDH served as an internal control. Data were expressed as mean ± SD from three independent experiments. (*Compared with control, ^#^ Compared with Mock + HG, */^#^*P* < 0.05, **/^##^*P* < 0.01)

The ROS level was tested by flow cytometry. Over-expression of FoxO1 was observed to sharply attenuate the ROS production by high glucose (Fig. 8c and d, *P* < 0.01). The expression of FoxO1 in nucleus was higher in HG + FoxO1 group than that in high glucose group. The expressions of FoxO1 in cytoplasm were increased in HG and HG + FoxO1 groups (Fig. 9a-b). The protein expressions of Catalase, MnSOD, NQO1 and HO1 showed a significant up-regulation in group over-expressing FoxO1 + HG compared to Mock + HG treatment (Fig. 9c and d, MnSOD, *P* < 0.05, Others, *P* < 0.01). The mRNA level of those four genes (Fig. 9e, MnSOD, *P* < 0.01, Others, *P* < 0.05) were consistent with proteins.

### Silencing FoxO1 reversed the effect of Stat1 silencing on apoptosis and oxidative stress

**Fig. 10:**
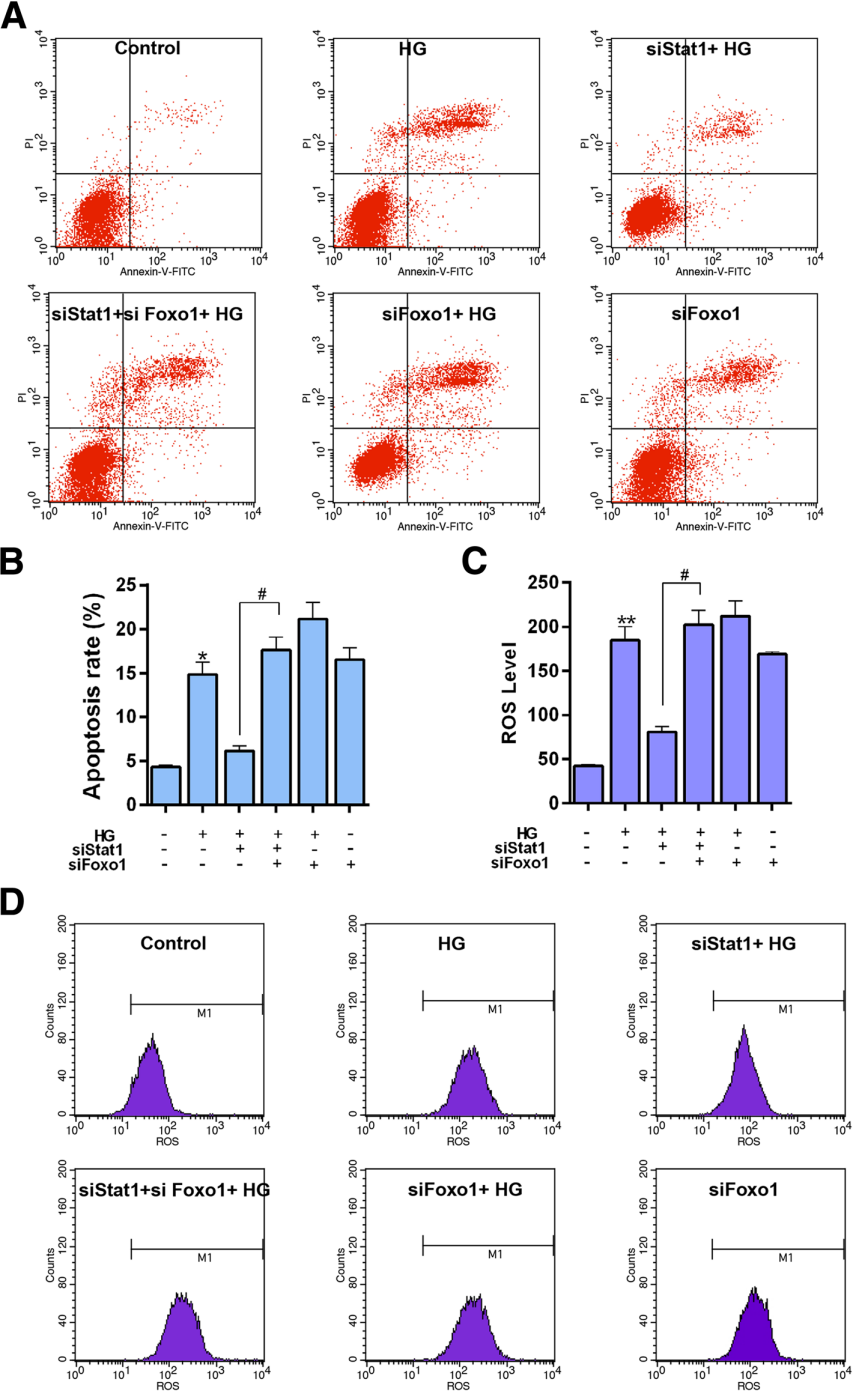
The effect of simultaneous silencing Stat1 and FoxO1 on apoptosis and ROS level. The experiments were divided into six groups, which were control, HG (30 μM), siStat1 + HG (Stat1 siRNA + 30 μM HG), siStat1 + siFoxO1 + HG (Stat1 siRNA + FoxO1 siRNA + 30 μM HG), siFoxO1 + HG (FoxO1 siRNA + 30 μM HG) and siFoxO1 (FoxO1 siRNA). **a** Apoptosis levels were detected by flow cytometry. **b** Relative apoptosis rate showed as bar diagrams. **c** ROS level showed as bar diagrams. **d** ROS levels were detected by flow cytometry. Data were expressed as mean ± SD from three independent experiments. (*Compared with control, ^#^ Compared with siStat1 + HG, */^#^*P* < 0.05, **/^##^*P* < 0.01)

In order to understand how silencing Stat1 protected high glucose-induced podocytes injury, we transfected silencing Stat1 and FoxO1 at the same time, and tested apoptosis level by flow cytometry. We found that simultaneous silencing Stat1 and Foxo1 attenuated the anti-apoptosis effect of silencing Stat1 (Fig. 10a and b, *P* < 0.05).

**Fig. 11:**
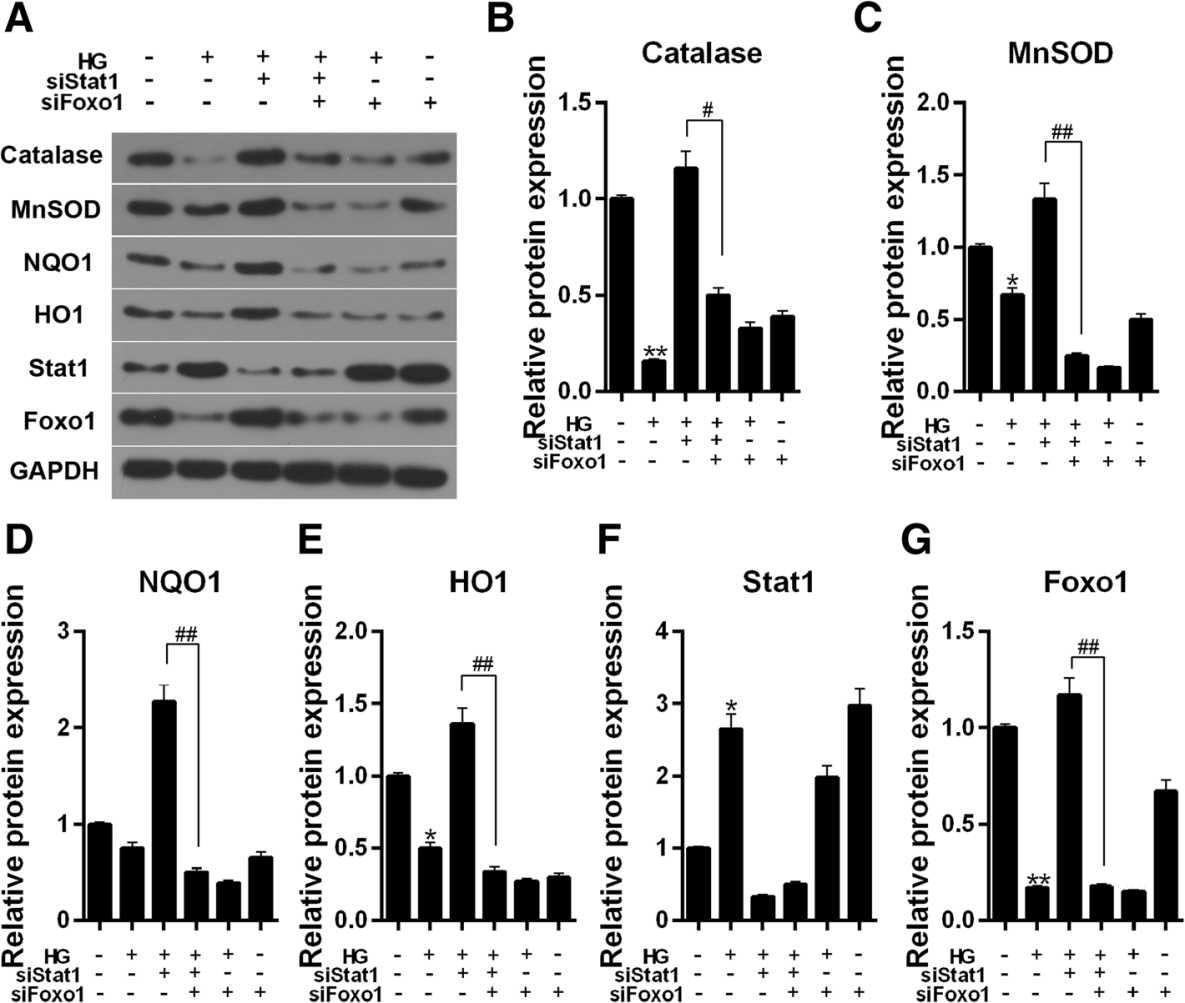
The effect of simultaneous silencing Stat1 and FoxO1 on the expression of antioxidant genes, Stat1 and FoxO1. The experiments divided were into six groups, which were control, HG (30 μM), siStat1 + HG (Stat1 siRNA + 30 μM HG), siStat1 + siFoxO1 + HG (Stat1 siRNA + FoxO1 siRNA + 30 μM HG), siFoxO1 + HG (FoxO1 siRNA + 30 μM HG) and siFoxO1 (FoxO1 siRNA). **a** The Catalase, MnSOD, NQO1, HO1, Stat1 and FoxO1 protein levels were detected by western blot in MPC-5 cells. **b** Relative protein expression of Catalase showed as bar diagrams. **c** Relative protein expression of MnSOD showed as bar diagrams. **d** Relative protein expression of NQO1 showed as bar diagrams. **e** Relative protein expression of HO1 showed as bar diagrams. **f** Relative protein expression of Stat1 showed as bar diagrams. **g** Relative protein expression of FoxO1 showed as bar diagrams. GAPDH served as an internal control. Data were expressed as mean ± SD from three independent experiments. (*Compared with control, ^#^ Compared with siStat1 + HG, */^#^*P* < 0.05, **/^##^*P* < 0.01)

ROS level increased with simultaneous silencing Stat1 and FoxO1 in HG-induced cells compared to silencing Stat1 (Fig. 10c and d, *P* < 0.05). Furthermore, the antioxidant genes (Catalase, MnSOD, NQO1, HO1) were also detected by western blot. Our results showed that Simultaneous silencing Stat1 and FoxO1 could significantly inhibited the protein and mRNA expressions of the antioxidant genes (Fig. 11a-e, Catalase, *P* < 0.05, Others, *P* < 0.01) compared to silencing Stat1 in HG induced cells.

## Discussion

Podocytes are highly differentiated cells with complex cytoskeletons [6]. They are the last barrier to glomerular filtration, and they play an important role in maintaining the integrity and function of the glomerular filtration barrier. Therefore, injury and apoptosis of podocytes would affect glomerular function [7, 33]. As previously reported in literature study, high glucose could cause podocytes injury [34–36].

The progression of DN is closely related to oxidative stress [11]. In this study, we used high glucose (30 mmol/L) to induce podocytes injury. Our results revealed that high glucose attenuated cell viability and promoted cell apoptosis. Stat1 is a member of a family of potential cytoplasmic transcription factors that are activated by cytokines and growth factors, which transmit signals from the cell surface to the nucleus [27]. Inhibition of Stat1 was involved in the antioxidant effect of SOCS1 in diabetes [37]. The expression of Stat1 was increased in patients with diabetic nephropathy [38], and the activated STAT1 was increased in high glucose-cultured mesangial cells (MCs) [39]. In addition, as one of the target genes of STAT1, FoxO1 plays a role in oxidant stress [40]. Thus we detected the protein expression of Stat1 and FoxO1. Our results showed that silencing Stat1 could significantly reverse the podocytes injury caused by high glucose. Specifically, siStat1 increased cell viability, inhibited cell apoptosis and attenuated ROS level in a high-glucose environment. Cleaved (active) caspase-3 is the executioner of apoptosis. The high glucose resulted in the increase of cleaved caspase-3 and Bax as well as the decreased Bcl-2. Interestingly, we found that mannitol suppressed the Bcl-2 at 48 h. however, we cannot explain the possible reason and it needs further study. Cleaved caspase-3 and pro-apoptosis protein Bax was found to be significantly down-regulated, and anti-apoptosis protein Bcl-2 was up-regulated by siStat1 compared to high glucose. Moreover, the expressions of Catalase, MnSOD, NQO1 and HO1 were increased by silencing Stat1. As two core antioxidant enzymes, Catalase and MnSOD were reported to be associated with elimination of ROS-mediated injuries [41]. MnSOD, a type of superoxide dismutase in eukaryotic cells [42], can eliminate O_2_ ¯· and plays an important role in anti-oxidation, retarding senility and wounding- resistant [43, 44]. As two important Phase II detoxification enzymes, NQO1 and HO-1 are downstream target genes regulated by Nrf2 [45]. NQO1 can be used as an electron transporter of NADH or NADPH to catalyze the reduction reaction of quinones and alleviate oxidative stress injury [46]. Stat family members could bind to FoxO1, and Stat1 played a negative role in FoxO1 promoter activity [47, 48]. We found that HG treatment decreased the Foxo1 expression in nucleus, and such a result was consistent with a research, which reported that Stat1 was a suppresser for FoxO1 promoter, and Stat1 can decrease the expression of FoxO1 [49]. The data also showed that Stat1 silencing could significantly increase the expression of FoxO1 in a high-glucose environment. Moreover, the overexpression of Foxo1 decreased the apoptosis and ROS generation generated by high glucose. In addition, the expressions of Catalase, MnSOD, NQO-1 and HO-1 were also higher in HG + Foxo1 group than those in HG group.

Simultaneous silencing Stat1 and FoxO1 were used in order to explore whether Stat1 protected podocytes from high glucose injury through Foxo1. We found that down-regulation of FoxO1 increased apoptosis, promoted ROS production and inhibited the expressions of Catalase, MnSOD, NQO-1 and HO-1 compared to siStat1. The results indicated that knockdown of Stat1 protected podocytes from high glucose injury via regulating the expression of FoxO1. Researchers reported that increased transcriptional activity of FoxO1 accounted for alteration in oxidative stress triggered by ROS accumulation [41, 50]. Moreover, low-level FoxO1 might be related to the reduced transcription of antioxidant enzymes [51]. Thus, our results were consistent with these studies.

## Conclusion

We explored the role of silencing Stat1 on high glucose-induced podocytes injury, and found that it could vreverse the effect of high glucose- triggered low cell viability, cell apoptosis and increased ROS production, and the functions of Stat1 was related to FoxO1 mediated-oxidative stress.

## Data Availability

The analyzed data sets generated during the study are available from the corresponding author on reasonable request.

## Abbreviations

ANOVA: Analysis of variance
CCK-8: Cell counting kit-8
DBD: DNA binding domain
DN: Diabetes nephropathy
FKH: Forkhead domain
GFB: Glomerular filtration barrier
HG: High glucose
MCs: Mesangial cells
NES: Nuclear export signal
NLS: Nuclear localization signal domain
NT: N-terminal oligomerization domain
OD: Optical density
PBS: Phosphate buffer saline
qRT-PCR: Quantitative real-time polymerase chain reaction
ROS: Reactive oxygen species
SD: Standard deviation
SH2: Src homology 2 domain
TA: Transactivation domain
TAD: Transcriptional activation region
